# The Mechanism of Hepatic Encephalopathy Induced by Thioacetamide Based on Metabolomics and Proteomics: A Preliminary Study

**DOI:** 10.3390/ijms25010284

**Published:** 2023-12-24

**Authors:** Honghui Guo, Guang Wang, Wei Huang, Lingrui Li, Yang Bai, Haifeng Wang, Lina Gao

**Affiliations:** 1 Liaoning Province Key Laboratory of Forensic Bio-Evidence Sciences, Shenyang 110122, China; 18856834146@163.com (H.G.); vivihuang98@163.com (W.H.);; 2 China Medical University Center of Forensic Investigation, Shenyang 110122, China; 3 Department of Forensic Analytical Toxicology, China Medical University, Shenyang 110122, China; 4 Department of Laboratory Animal Science, China Medical University, Shenyang 110122, China; gwang@cmu.edu.cn

**Keywords:** thioacetamide, proteomics, metabolomics, ferroptosis, hepatic encephalopathy

## Abstract

Hepatic encephalopathy (HE) is a central nervous system dysfunction syndrome caused by acute and chronic liver failure or various portal systemic shunt disorders. HE arises from metabolic disorder and excludes other known types of encephalopathy. HE is a major cause of death in people with liver disease. Early diagnosis and timely treatment are key to improving HE prognosis. Herein, we established a model of HE and performed metabolomics to identify 50 significantly differential metabolites between the HE group and control group. The main metabolic pathways associated with these differential metabolites were the purine metabolism, pyrimidine metabolism, aminoacyl tRNA biosynthesis, and glucose metabolism. Through proteomics analysis, we identified 226 significantly differential proteins (52 up-regulated and 174 down-regulated). The main (Kyoto Encyclopedia of Genes and Genomes) enrichment pathways were the *Staphylococcus aureus* infection, vitamin digestion and absorption, and complement and coagulation cascades. Through the conjoint analysis of proteomics and metabolomics, the differentially present proteins and metabolites were found to be involved in vitamin digestion and absorption, and ferroptosis pathways. In HE, malondialdehyde was significantly elevated, but glutathione was significantly diminished, and the redox balance was destroyed, thus leading to changes in proteins’ levels associated with the ferroptosis pathway. In conclusion, this study preliminarily explored the molecular and metabolic mechanisms underlying HE.

## 1. Introduction

Hepatic encephalopathy (HE) is a syndrome of central nervous system dysfunction caused by acute and chronic liver failure or various portal systemic shunt disorders; it is based on metabolic disorder and excludes other known types of encephalopathy [1]. HE is the third most serious complication of decompensated liver cirrhosis and affects at least 30% of people with cirrhosis [2]. HE is characterized by a series of neuropsychiatric syndromes as diverse as subclinical changes and coma, including abnormal changes in cognitive function, emotion, behavior, and motor function. Clinically, HE is divided primarily into type A, type B, and type C. Type A is caused by acute liver failure, which is characterized by an acute onset, a long course of disease, and a mortality rate as high as 80%. The pathological basis of HE is complex, and its pathogenesis is not yet clear. Beyond the ammonia poisoning theory [3], inflammatory cytokines [4,5,6,7], neurosteroids [8,9,10], endogenous benzodiazepines [11], bile acids [12,13], and manganese [8] may participate in the occurrence of HE. In recent years, many researchers have found that the influence of ammonia on neurotransmitters plays an important role in the pathogenesis of HE [14,15]. In 2000, Butterworth [16] reported that the primary cause of HE is neurotransmitter abnormalities rather than energy failure. Jones [17] has clearly shown that the occurrence of HE is due to an imbalance between inhibitory neurotransmitters (γ-aminobutyric acid (GABA)) and excitatory neurotransmitters (glutamate), thus leading to a net increase in inhibitory neurotransmitters. The molecular and metabolic mechanisms of HE have not been fully elucidated, and current clinical interventions primarily decrease the formation and absorption of ammonia. On the basis of the pathogenesis of HE, the clinical application of non-absorbable disaccharides, rifaximin, and L-ornithine-L-aspartate (LOLA) is used to decrease circulating ammonia. Probiotics and fecal microbiota transplantation have been used to modulate the gut microbiota composition and ameliorate gastrointestinal disorders; other treatments such as human albumin therapy and branched chain amino acid therapy are also commonly applied [18,19,20,21,22,23,24,25]. To develop more effective treatments and therapies, basic research on the pathogenesis of HE is essential and urgently needed.

An experimental model of HE using thioacetamide (TAA) in mice has been validated [26,27] and involves a dose of 200 mg/kg, injected intraperitoneally for 3 consecutive days, thus achieving a final dose of 600 mg/kg [28,29]. The model mimics human acute progressive hepatic disorders with the concomitant involvement of the brain [30]. TAA is extensively metabolized to very reactive metabolites that bind liver macromolecules, thus resulting in hepatic necrosis, hyper-ammonemia [31], and extensive oxidative stress [32].

Proteomic methods such as high-resolution mass spectrometry are used to study protein composition and changes in cells, tissues, or organisms. The study of the proteome can provide a theoretical basis and solutions for elucidating many disease mechanisms. Through the comparative analysis of the proteome between normal individuals and those with pathologies, disease-specific protein molecules can be identified. Tandem mass tag technology, developed by Thermo Scientific (Waltham, MA, USA) [33], enables relative and absolute quantification, and is often used in quantitative proteomics. Metabolomics, a component of systems biology, is a research method applying genomics and proteomics to quantitatively analyze all metabolites in any organism, and determine the relationships of metabolites with both physiological and pathological changes.

Herein, we sought to explore the mechanism of HE induced by TAA by using metabolomics and proteomics. To our knowledge, we report the first joint analysis of proteomics and metabolomics initially revealing the molecular and metabolic mechanisms of HE.

## 2. Results

### 2.1. H&E Staining

**Figure 1:**
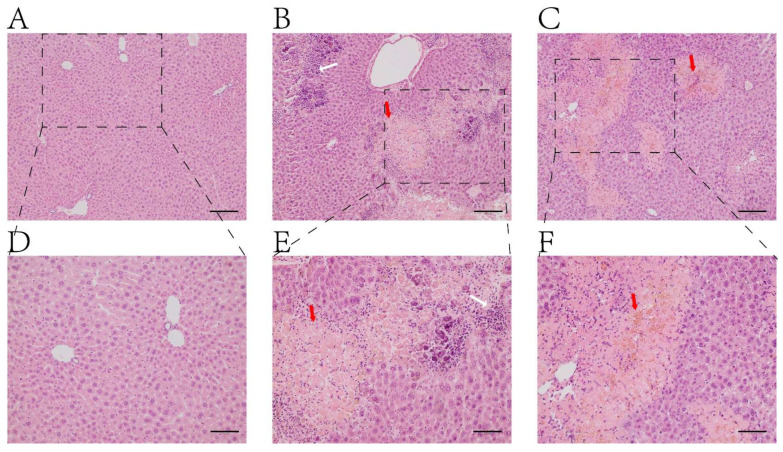
Histopathological examinations of H&E stain of liver. (**A**,**D**): Normal group; (**B**,**C**,**E**,**F**): TAA-induced model group. (**A**–**C**): 100× magnifications, bar = 100 µm; (**D**–**F**): 200× magnifications, bar = 50 μm. White arrow: infiltrations of inflamed cells; Red arrow: Ballooning degeneration and hepatic sinus congestion.

The pathological results of the liver in mice in each group are shown in Figure 1. The liver lobular structure of mice in the normal group was normal, and there was no degeneration or necrosis of the liver cells. The mild and moderate watery degeneration of hepatocytes was observed in the experimental group. There was obvious hepatocyte lysis and necrosis, inflammatory cell infiltration, and lobular structure disorder in the necrotic area.

**Figure 2:**
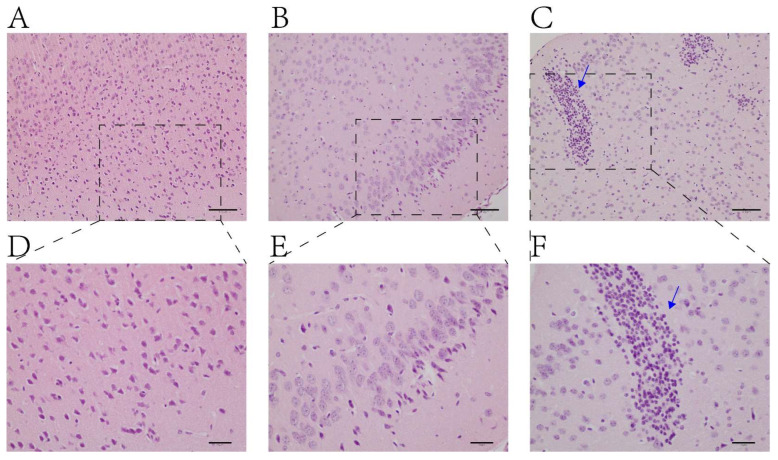
Histopathological examinations of H&E stain of brain. (**A**,**D**): Control group; (**B**,**C**,**E**,**F**): TAA-induced model group. (**A**–**C**): 200× magnifications, bar = 50 μm; (**D**–**F**): 400× magnifications, bar = 20 μm. Blue arrow: Glial cells proliferate and accumulate.

The brain pathological results of mice in each group are shown in Figure 2. The cell structure of the brain tissue of mice in the normal group was normal and complete with a full nucleus, while the cell structure of the brain tissue of mice in the experimental group was disordered with glial cell hyperplasia.

### 2.2. Results of ALT, AST, GSH, and MDA

**Figure 3:**
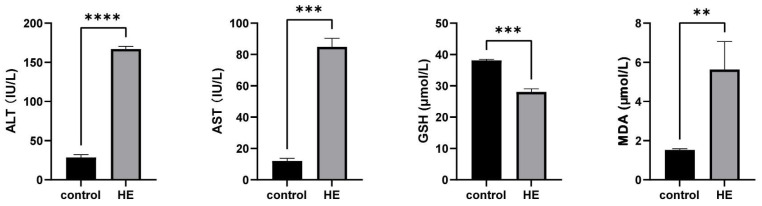
The effects of thioacetamide on mouse serum biochemistry. The levels of ALT, AST, GSH, and MDA in serum of mice are shown. AST and ALT are important indicators of liver function, and their simultaneous elevation indicates liver cell damage and abnormal liver function. The decrease of GSH indicates the occurrence of oxidative damage. Lipid peroxidation as determined by MDA content. Values shown are means ± SEM; The symbols show the following significances: *p* < 0.01 = **; *p* < 0.001 = ***; *p* < 0.0001 = ****.

As shown in Figure 3, compared with the control group, the levels of ALT (alanine transaminase) and AST (aspartate aminotransferase) in the serum of mice in the HE group were significantly increased (*p* ≤ 0.01), indicating the liver injury of mice after modeling. Compared with the control group, the content of GSH in the serum of the model group decreased significantly (*p* ≤ 0.01), and the activity of MDA increased significantly.

### 2.3. Results of Metabolomics

#### 2.3.1. Sample Quality Control

The Pearson correlation coefficient between QC samples is calculated based on the relative quantitative value of metabolites. The higher the correlation of QC samples (the closer R2 is to 1), the better the stability of the whole detection process and the higher the data quality. The correlation of QC samples is shown in Supplementary Figure S1.

#### 2.3.2. PCA and PLS-DA Results

**Figure 4:**
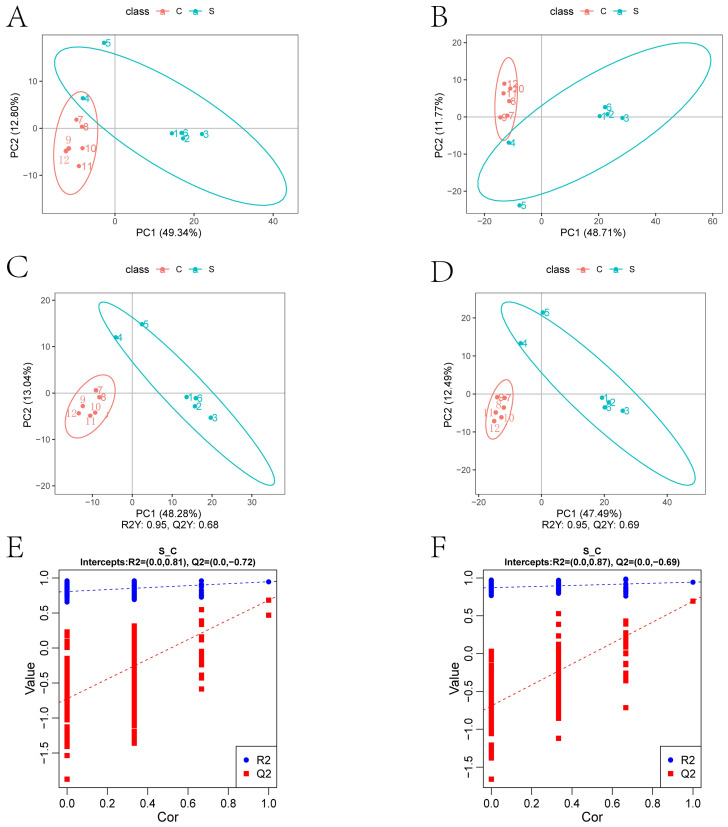
Metabolic profile of mouse brain tissue (**A**,**B**): PCA (principal component analysis); (**C**,**D**): Score plot of the partial least squares discriminant analysis (PLS-DA): abscissa is the score of the sample in the first principal component, the ordinate is the score of the sample in the second principal component, R2Y represents the interpretation rate of the model, Q2Y was used to evaluate the predictive power of the PLS-DA model, and when R2Y is greater than Q2Y, the model is well established; (**E**,**F**): Sort validation graph of Partial least squares discriminant analysis, R2 displays the variance explained in the model and indicates the goodness of fit. Q2 displays the variance in the data predictable by the model and indicates the predictability. In each part, the left one was obtained in the negative polarity mode, and the right one was obtained in the positive polarity mode.

We found that the HE group was significantly different from the control group through principal component analysis (PCA) (as shown in Figure 4A,B) and partial least squares discrimination analysis (PLS-DA) (as shown in Figure 4C,D). We tested whether the model was “overfitted”. Whether the model is “overfitted” reflects the accuracy of the model construction. Failure to be “overfitted” indicates that the model can better describe the sample. These results are shown in Figure 4E,F.

#### 2.3.3. Volcano Plots and Hierarchical Clustering Analysis of Differential Metabolites

**Figure 5:**
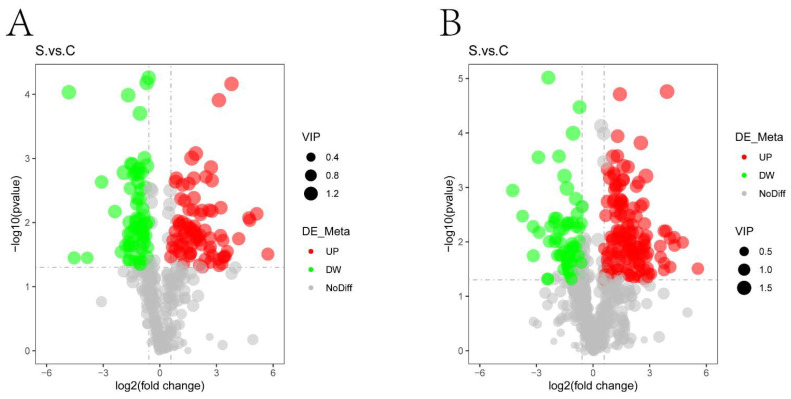
Volcano plots and metabolite heatmaps of differential metabolites. (**A**,**B**): Volcano plots of differential metabolites in (**A**) negative and (**B**) positive polarity mode. Significant difference for the *p* value on vertical ordinate (Base 10 logarithmic transformation). Red represents up-regulation, green represents down-regulation in the HE group, gray represents that there is no distinguished difference between the HE group and the control group, and VIP represents the importance projection value of this substance obtained in the PLS-DA model between different groups.

**Figure 6:**
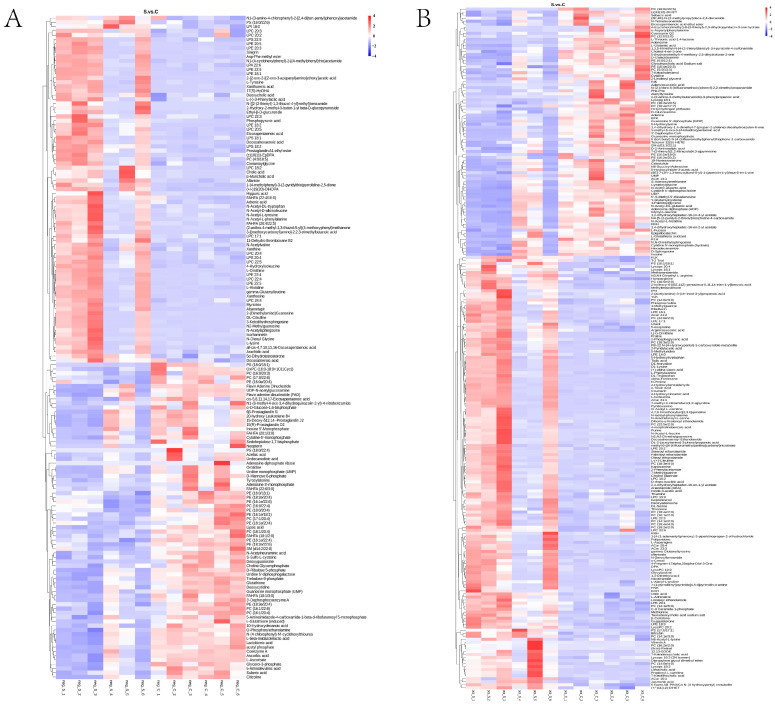
Metabolite heatmaps of differential metabolites. (**A**,**B**): The heatmaps of the metabolites between different groups in (**A**) negative and (**B**) positive polarity mode.

The volcano plot can directly show the overall distribution of differential metabolites, the abscissa represents the difference fold change of metabolites in different groups (log_2_FoldChange), the ordinate indicates the difference significance level (−log10*p*-value), each point in the volcano plot represents a metabolite, significantly up-regulated metabolites are represented by red dots, significantly down-regulated metabolites are represented by green dots, and the size of the dot represents the VIP value, as shown in Figure 5. Hierarchical clustering analysis of the obtained differential metabolites was performed to obtain the differences in the metabolic expression patterns between and within two groups for the same comparison, as shown in Figure 6. For a more intuitive view of differential metabolites, we listed significantly divergent representative metabolites, as shown in Table 1.

#### 2.3.4. KEGG Pathway Enrichment Analysis

**Figure 7:**
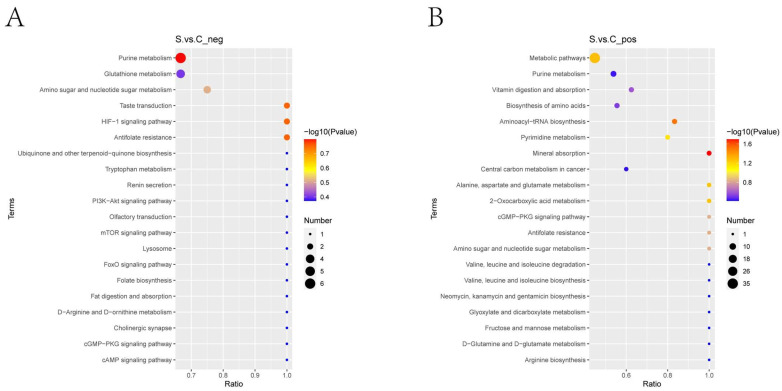
The KEGG pathway that the distinguished metabolites take part in between the HE group and the control group. Terms in the same category were ranked based on the *p*-values. (**A**): the negative polarity mode; (**B**): the positive polarity mode. The abscissa in the figure is the ratio of the number of different metabolites in the corresponding pathway to the number of identified total metabolites. The higher the ratio, the higher the concentration of differential metabolites in the pathway. The color of the dot represents the *p*-value of the hypergeometric test. The smaller the *p*-value, the greater the reliability and the more statistically significant the test. The size of the dot represents the quantity of differential metabolites in the corresponding pathway. A larger point size indicates more different metabolites in the pathway. S represents the HE group; C represents the control group.

The differential metabolites were also enriched for the KEGG metabolic pathways; the enrichment KEGG pathways are the purine metabolism, pyrimidine metabolism, aminoacyl-tRNA biosynthesis, and glutathione metabolism, as shown in Figure 7.

### 2.4. Results of Proteomics

#### 2.4.1. Differential Protein Results for Presentation, Volcano Plots, and Heatmap Plots

Protein difference analysis first selects the sample pairs that need to be compared, and takes the ratio of the mean of all biological repeated quantitative values of each protein in the comparison sample pair as the fold change (FC). In order to judge the significance of the difference, the relative quantitative value of each protein in the two comparison samples was tested by a t-test, and the corresponding *p*-value was calculated as the significance index, with the default *p*-value ≤ 0.05. The number of up- and down-regulated proteins screened according to this condition is shown in Supplementary Table S1. As a result, we found 52 up-regulated proteins and 174 down-regulated proteins.

**Figure 8:**
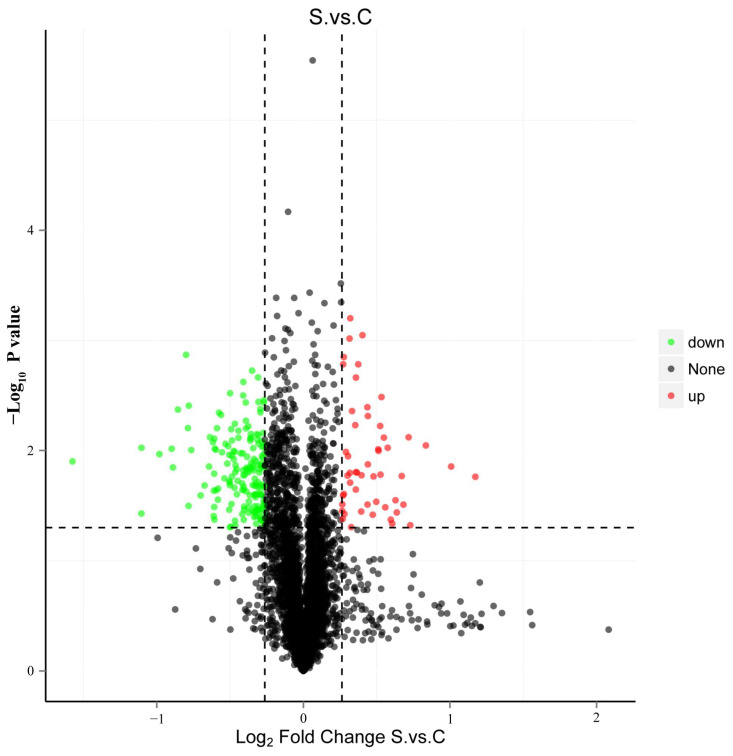
Volcano plots of proteins with differential expression (FC > 1.2 or FC < 0.83, *p* value < 0.05). Significant difference for the *p* value on vertical ordinate (Base 10 logarithmic transformation). Red dots represent up-regulated, green dots represent down-regulated, and black dots represent no significant difference. S represents the HE group; C represents the control group.

**Figure 9:**
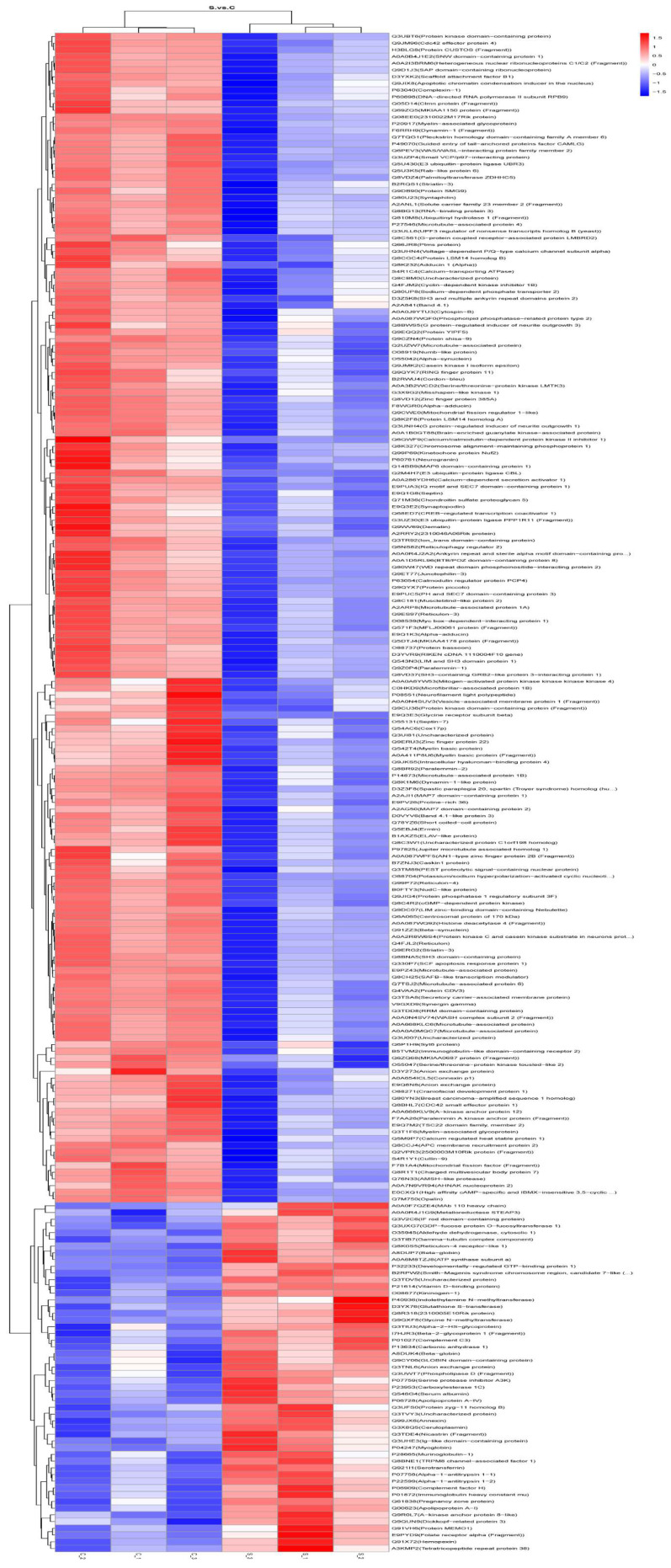
The heatmaps of DEPs between the HE group and the control group. Red represents up-regulation; blue represents down-regulation. S represents the HE group; C represents the control group.

Differentially expressed proteins (DEPs) are shown by a volcano plot and heatmap, as shown in Figure 8 and Figure 9. DEPs are displayed in a volcano graph, where the black dots represent indifferent proteins, and the up-regulated and down-regulated proteins are represented by red and green dots, respectively.

Cluster analysis was carried out for the relative content of DEPs in each sample and the up-regulation and down-regulation of DEPs among the comparison groups were observed in the cluster analysis of DEPs. When the fold change (FC) ≥ 1.2 and the *p*-value < 0.05, up-regulated expressed proteins were screened. When the FC ≤ 0.83 and the *p*-value < 0.05, down-regulated expressed proteins were screened.

#### 2.4.2. DEPs of Gene Ontology (GO) Function Enrichment Analysis

**Figure 10:**
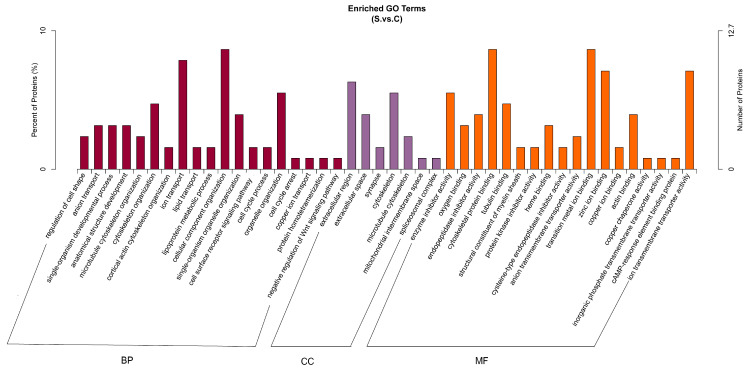
Gene ontology (GO) term (including biological process, cellular component, and molecular function) enrichment for differentially expressed proteins between the HE group and the control group. BP: biological Process, CC: Cellular Component, MF: Molecular Function. The left ordinate represents the number of detected differential proteins associated with the GO as a percentage of the number of differential proteins annotated by GO, and the right ordinate represents the number of detected differential proteins associated with the GO. S represents the HE group; C represents the control group.

Bioinformatics GO enrichment analysis can indicate what biological functions are significantly related to DEPs. As shown in Figure 10, compared with the control group, the related biological processes of the screened DEPs in the HE group were ion transport, cellular component organization, cytoskeleton organization, organelle organization, etc. These DEPs are mainly located in the extracellular region and cytoskeleton and their main molecular functions were transition metal ion binding, cytoskeletal protein binding, ion transmembrane transporter activity, and zinc ion binding, etc.

#### 2.4.3. Enrichment of Kyoto Encyclopedia of Genes and Genomes (KEGG) Pathway Analysis

**Figure 11:**
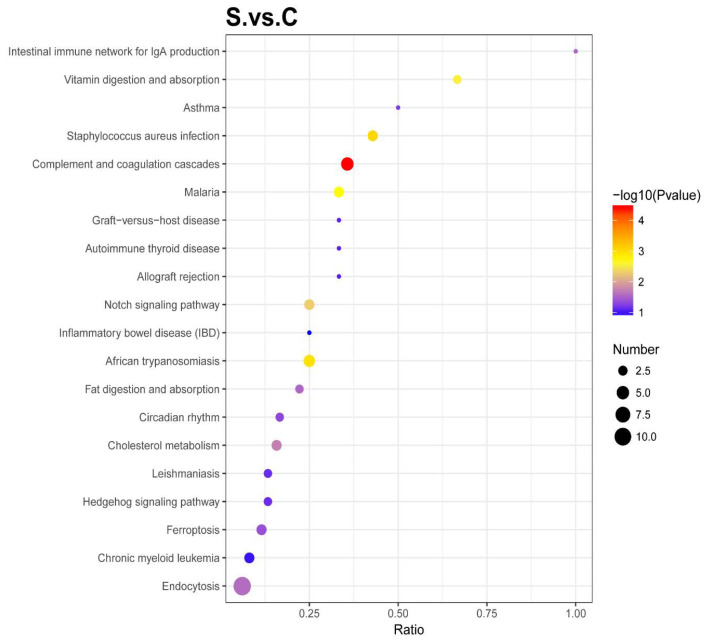
Enrichment KEGG pathway. The abscissa represents the ratio of the number of differential proteins associated with the pathway to the number of background (all) proteins associated with the pathway. The redder the bubble represents the smaller the *p* value, the bluer the bubble represents the larger the *p* value, and the larger the bubble represents the more differential proteins detected. (Top 20 between the HE group and the control group; S represents the HE group; C represents the control group.)

The significant enrichment of a pathway can determine the main biochemical metabolic and signal transduction pathways involved in DEPs, as shown in Figure 11. According to the enrichment results, the enriched KEGG pathways were plotted (only the results of the Top 20 are shown). Compared with the control group, the main enriched pathways in the HE group were complement and coagulation cascades, vitamin digestion, and absorption.

### 2.5. Joint Analysis of Metabolomics and Proteomics

**Figure 12:**
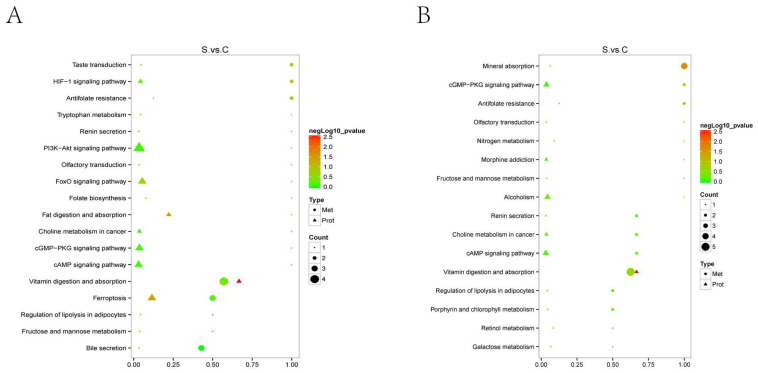
Joint analysis of metabolomics and proteomics. The abscissa in the figure is the ratio of the number of differential metabolites or DEPs in the corresponding pathway to the number of identified total metabolites or proteins. The color of the dot represents the *p*-value of the hypergeometric test. The smaller the *p*-value, the greater the reliability and the more statistically significant the test. The size of the dot represents the quantity of differential metabolites or DEPs in the corresponding pathway. Triangle represents DEPs, Circles represent differential metabolites. S represents the HE group; C represents the control group. (**A**): the negative polarity mode; (**B**): the positive polarity mode.

Through the joint analysis of metabolomics and proteomics, as shown in Figure 12, the main enrichment pathways are vitamin digestion and absorption and the ferroptosis pathway. The metabolites and proteins related to vitamin digestion and absorption are pyridoxamine, thiamine, nicotinamide, vitamin A, riboflavin, and aldehyde dehydrogenase. The metabolites and proteins related to the ferroptosis pathway are adrenic acid, coenzyme A, glutathione, STEAP family member 3, and ceruloplasmin serotransferrin.

### 2.6. Detect Ferroptosis-Related Gene Expression in Mouse Brain Tissue

**Figure 13:**
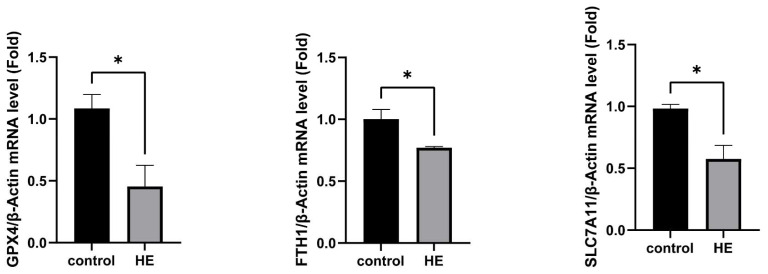
Quantification of relative mRNA expression normalized to β-actin. Ferroptosis-related mRNA expression is decreased in HE group. The relative mRNA levels of GPX4, FTH1, and SLC7A11 in mouse brain tissue injected with or without thioacetamide. Values shown are means ± SEM; The symbols show the following significances: *p* ≤ 0.05 = *.

A range of ferroptosis-related gene expressions, including GPX4 (Glutathione peroxidase 4), was examined. GPX4, SLC7A11, and FTH1 mRNA levels (Figure 13) were dramatically decreased in mice of thioacetamide treatment.

## 3. Discussion

In this study, the TAA induction method was used to establish a model of acute HE. TAA is metabolized by cytochrome P450 oxidase in liver cells, thus producing toxic TAA sulfur oxide, and subsequently causing lipid peroxidation, liver metabolic disorder, and other damage. Subsequently, inflammatory cells infiltrate the liver tissue, and hepatocyte necrosis and other changes occur; finally, acute liver failure induces type A HE. In this study, liver biochemical indicators and brain tissue staining in the HE group significantly differed from those in the control group, thus indicating the successful establishment of a mouse model of acute HE.

Applying metabolomics, we selected 50 differential metabolites between the HE group and the control group. These metabolites were concentrated primarily in the up-regulation of amino acids (including threonine, citrulline, isoleucine, lysine, proline, ornithine, tryptophan, and phenoylalanine). Some differential metabolites were involved in energy metabolism pathways (down-regulated metabolites included adenine, adenosine, cytidine, uridine diphosphate N-acetylglucosamine, cytidine 5′-monophosphate, inosine, adenosine 5′-monophosphate, uridine monophosphate, guanosine monophosphate, and glucose-1,6-bisphosphate; up-regulated metabolites included N2-methylguanosine, xanthosine, uracil, and argininosuccinic acid) and the reduction of antioxidant properties such as ascorbic acid and glutathione. Glutathione maintains the redox balance in the body. In the HE group, metabolites related to glutathione, such as γ-glutamylcysteine, S-adenosylmethionine, and L-glutathione, were all significantly down-regulated in the HE group. By measuring the content of GSH and MDA, we determined that TAA causes oxidative stress damage to brain and liver tissues. This finding was consistent with the TAA-induced lipid peroxidation and oxidative damage reported by Essam et al. [34]. In addition, metabolites associated with inflammation, such as L-histidine and leukotriene B4, were also significantly up-regulated in the HE group, thus indicating that the inflammatory response played an important role in the pathological process of HE [35,36]. Large amounts of inflammatory mediators and high levels of blood ammonia synergistically affected the brain, thereby triggering inflammatory reactions in the central nervous system.

In the HE group, compared with the control group, GABA was higher and glutamic acid was lower, in agreement with previous findings indicating that HE is associated with neurotransmitter disorder [9]. The roles of abnormal neurotransmitters in the pathogenesis of HE have attracted substantial research attention. Jones has reported that HE occurs because of an imbalance between inhibitory neurotransmitters such as GABA and excitatory neurotransmitters such as glutamate [11].

Proteomic analysis indicated that the functions of DEPs were concentrated in complement and coagulation cascades, and vitamin digestion and absorption. Some studies have also shown that complement and coagulation cascade pathways are involved in degenerative disease [37]. The vitamin digestion and absorption pathways arose from changes in metabolites (such as thiamine, nicotinamide, and riboflavin) and proteins (aldehyde dehydrogenase) in the HE group compared with the control group.

According to a joint proteomic and metabolomic analysis, the main KEGG pathways in the TAA-induced HE model were the HIF-1 signaling pathway, PI3K-Akt signaling pathway, and ferroptosis pathway. Previous studies have indicated that the HIF-1 signaling pathway is involved in molecular mechanisms in liver fibrosis models [38]. Some studies have reported that the PI3K-Akt signaling pathway contributes to the brain molecular mechanism of HE [39,40]. In this study, the joint analysis demonstrated that the changes in the ferroptosis pathway involved the small molecule glutathione, and the proteins six-transmembrane epithelial antigen of prostate 3 (STEAP3), ceruloplasmin, and serotransferrin in the HE group. STEAP3, a key regulator of iron uptake, is involved in immunity and apoptotic processes in various cell types [41,42]. Studies have shown that this protein participates in the regulation of liver ischemia-reperfusion injury, hepatocellular carcinoma, myocardial hypertrophy, and other diseases. Moreover, Ding et al. [41] have found that the expression of STEAP3 is up-regulated in nonalcoholic fatty liver disease. Furthermore, Guo et al. have reported that STEAP3 knockout protects the liver against ischemia-reperfusion injury [42]. In the present study, STEAP3 was found to be up-regulated in the HE group, thus suggesting its involvement in this syndrome. In the HE model, up-regulated serotransferrin suggested that iron metabolism was affected. These findings suggested that the ferroptosis pathway may play a role in the TAA-induced HE model.

According to quantitative RT-PCR analysis, GPX4, SLC7A11 and FTH1 mRNA levels were dramatically diminished in the HE group. Glutathione peroxidase 4 (GPX4) is a recognized ferroptosis gatekeeper with a central role in limiting lipid peroxidation [43]. GPX4 uses glutathione to protect cells against ferroptosis by eliminating phospholipid peroxides [44]. The brain has the second highest concentration of lipids among the diverse organs of the human body; moreover, the high oxygen consumption rate of the brain makes this organ particularly vulnerable and sensitive to lipid peroxidation [45]. GPX4 is the most widely expressed GPX isoform in the brain, and it acts as a powerful antioxidant [46]. GPX4 expression has been found to be down-regulated in many brain diseases [47,48].

**Figure 14:**
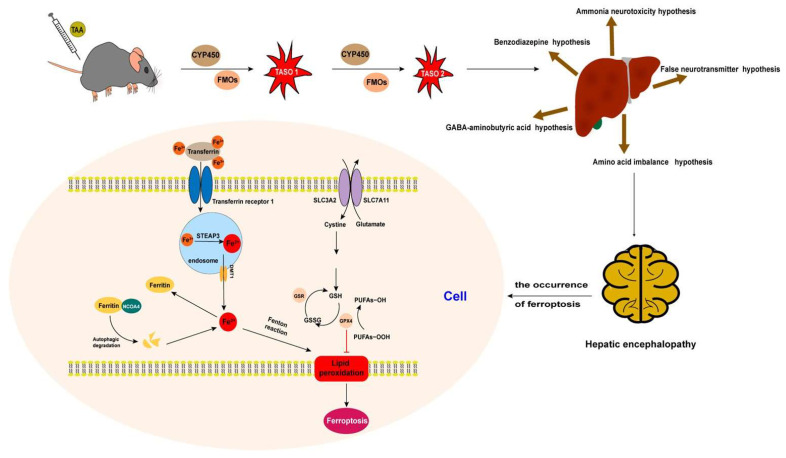
Hepatic encephalopathy after intraperitoneal injection of thioacetamide may be associated with ferroptosis.

SLC7A11 and FTH1 also play important roles in many brain diseases associated with ferroptosis. SLC7A11, the cystine/glutamate antiporter involved in GSH synthesis, neutralizes oxidative substances in the cell membrane [49]. FTH1 has ferroxidase activity, catalyzing the conversion of ferrous (Fe^2+^) to ferric (Fe^3+^) iron for storage in ferritin nanocages, thereby attenuating the iron-mediated catalysis of reactive oxygen species. In our study, GPX4, SLC7A11, and FTH mRNA expression was significantly down-regulated in the HE group, thus suggesting the involvement of ferroptosis in our model of TAA-induced HE. As shown in Figure 14, TAA-induced hepatocyte injury leading to HE may be associated with ferroptosis in brain tissue.

## 4. Materials and Methods

### 4.1. Reagents

Liquid chromatography–Mass spectrometry (LC-MS)-grade ultrapure water, LC-MS-grade acetonitrile, and LC-MS-grade formic acid were purchased from Thermo Fisher Chemical (Waltham, MA, USA). Acetone was purchased from Beijing Chemical Works Co., Ltd. (Beijing, China). ProteoMiner Low Abundance Protein Enrichment Kit was purchased from Bio-Rad (Hercules, CA, USA). LC-MS/MS analysis was performed by Novogene Co., Ltd. (Beijing, China). Aspartate aminotransferase (AST) enzyme-linked immunosorbent assay (ELISA) and alanine aminotransferase (ALT) ELISA kits were purchased from Shanghai Enzyme-linked Biotechnology Co., Ltd. (Shanghai, China). GSH and SOD Biochemical Assay Kit were purchased from Elabscience Biotechnology Co., Ltd. (Wuhan, China). Liquid chromatography–mass spectrometry (LC–MS)-grade water, acetonitrile (ACN), Methanol (MeOH), and formic acid (FA) were purchased from Thermo Fisher Scientific (Waltham, MA, USA). LC–MS/MS analysis was performed by Novogene Co., Ltd. (Beijing, China). AG RNAex Pro Reagent, Evo M-MLV RT Mix Kit with gDNA Clean for Qpcr, and SYBR^®^ Green Premix Pro Taq HS qPCR Kit were purchased from Accurate Biotechnology(Human)Co., Ltd. (Changsha, China).

### 4.2. Animal Model and Sample Collection

All animal experiments were approved by the Animal Ethics Committee of China Medical University and performed according to the National Institutes of Health guide for the care and use of Laboratory animals (NIH Publications No. 8023, revised 1978) and to the Guidelines for the Care and Use of Laboratory Animals of China Medical University (ethical approval code is KT2022353). Six-week-old, specific pathogen-free (SPF)-grade male Kunming mice (KM mice, originated from Swiss mice, the largest outbred group in China and widely used in pharmacology, toxicology, and other fields of research) weighing 30 ± 5 g were obtained from Beijing Huafukang Biotechnology Co., Ltd. (Beijing, China). A total of 12 KM mice were randomly divided into the HE groups and the control group (*n* = 6 in each group). The HE group were given thioacetamide by intraperitoneal injection at the dose of 200 mg/kg at three alternative days. The control group was injected with normal saline every other day, and all were sacrificed on the sixth day of the experiment. At the end of the experiment, the mice were euthanized via compressed gas in their home cage by trained personnel. Death was confirmed after checking for lack of respiration and faded eye color in each mouse. The brain was excised and the cerebrum quickly separated. Serum samples were stored at −80 °C for detection of GSH activity and MDA content. Parts of the cerebral cortex were homogenized in cold phosphate-buffered saline (PBS), centrifuged, and the clear homogenate was collected for biochemical assays.

### 4.3. Proteomics

#### 4.3.1. Total Protein Extraction

The cerebrum was ground individually in liquid nitrogen and lysed with PASP lysis buffer (100 mM NH_4_HCO_3_, 8 M Urea, pH 8), followed by 5 min of ultrasonication on ice. The lysate was centrifuged at 12,000× *g* for 15 min at 4 °C and the supernatant was reduced with 10 mM DTT for 1 h at 56 °C, and subsequently alkylated with sufficient IAM for 1 h at room temperature in the dark.

Then samples were completely mixed with 4 times volume of precooled acetone by vortexing, and incubated at −20 °C for at least 2 h. Samples were then centrifuged at 12,000× *g* for 15 min at 4 °C and the precipitation was collected. After washing with 1 mL cold acetone, the pellet was dissolved by dissolution buffer (8 M Urea, 100 mM TEAB, pH 8.5).

#### 4.3.2. TMT Labeling of Peptides

Each protein sample was taken and the volume was made up to 100 μL with DB dissolution buffer (8 M Urea, 100 mM TEAB, pH 8.5). Trypsin and 100 mM TEAB buffer were added, and sample was mixed and digested at 37 °C for 4 h. Then, trypsin and CaCl_2_ were added, and sample was digested overnight. Formic acid was mixed with digested sample, adjusted pH under 3, and centrifuged at 12,000× *g* for 5 min at room temperature. The supernatant was slowly loaded to the C18 desalting column, washed with washing buffer (0.1% formic acid, 3% acetonitrile) 3 times, then eluted by some elution buffer (0.1% formic acid, 70% acetonitrile). The eluents of each sample were collected and lyophilized. Next, 100 μL of 0.1 M TEAB buffer was added to reconstitute, and 41 μL of acetonitrile-dissolved TMT labeling reagent was added; sample was mixed with shaking for 2 h at room temperature. Then, the reaction was stopped by adding 8% ammonia. All labeling samples were mixed with equal volume, desalted, and lyophilized.

#### 4.3.3. Separation of Fractions

Mobile phase A (2% acetonitrile, adjusted pH to 10.0 using ammonium hydroxide) and B (98% acetonitrile) were used to develop a gradient elution as shown in Table 2. The lyophilized powder was dissolved in solution A and centrifuged at 12,000× *g* for 10 min at room temperature. The sample was fractionated using a C18 column (Waters BEH C18, 4.6 × 250 mm, 5 μm) on a Rigol L3000 HPLC system and the column oven was set as 45 °C. The eluates were monitored at UV 214 nm, collected for a tube per minute and combined into 10 fractions finally. All fractions were dried under vacuum and then reconstituted in 0.1% (*v*/*v*) formic acid in water.

#### 4.3.4. LC-MS/MS Analysis

For transition library construction, shotgun proteomics analyses were performed using an EASY-nLCTM 1200 UHPLC system (Thermo Fisher) coupled with a Q ExactiveTM HF-X mass spectrometer (Thermo Fisher) operating in the data-dependent acquisition (DDA) mode. For this, 1 μg sample was injected into a home-made C18 Nano-Trap column (4.5 cm × 75 μm, 3 μm). Peptides were separated in a home-made analytical column (15 cm × 150 μm, 1.9 μm) using a linear gradient elution, as listed in Table 3. The separated peptides were analyzed by Q Exactive TM HF-X mass spectrometer (Thermo Fisher), with ion source of Nanospray Flex™ (ESI), spray voltage of 2.1 kV, and ion transport capillary temperature of 320 °C. Full scan range was from *m*/*z* 350 to 1500 with resolution of 60,000 (at *m*/*z* 200), an automatic gain control (AGC) target value was 3 × 10^6^, and a maximum ion injection time was 20 ms. The top 40 precursors of the highest abundant in the full scan were selected and fragmented by higher energy collisional dissociation (HCD) and analyzed in MS/MS, where resolution was 30,000 (at *m*/*z* 200) for 6 plex, the automatic gain control (AGC) target value was 5 × 104, the maximum ion injection time was 54 ms, a normalized collision energy was set as 32%, an intensity threshold was 1.2 × 10^5^, and the dynamic exclusion parameter was 20 s. The raw data of MS detection were named “.raw”.

#### 4.3.5. Data Analysis

##### The Identification and Quantitation of Protein

The resulting spectra from each run were searched separately against Mus_musculus_uniprot_2021_7_15.fasta (86,544 sequences) by the search engines: Proteome Discoverer 2.4 (PD 2.4, Thermo). The searched parameters were set as follows: Mass tolerance for precursor ion was 10 ppm and mass tolerance for product ion was 0.02 Da. Carbamidomethyl was specified as fixed modifications, Oxidation of methionine (M) and TMT plex were specified as dynamic modification. Acetylation, TMT plex, Met-loss. and Met-loss+Acetyl were specified as N-Terminal modifications in PD 2.4. A maximum of 2 missed cleavage sites were allowed.

In order to improve the quality of analysis results, the software PD 2.4 further filtered the retrieval results: Peptide Spectrum Matches (PSMs) with a credibility of more than 99% were identified PSMs. The identified protein contained at least 1 unique peptide. The identified PSMs and protein were retained and performed with FDR of no more than 1.0%. The protein quantitation results were statistically analyzed by T-test. The proteins whose quantitation significantly differed between experimental and control groups (*p* < 0.05 and |log_2_FC| > 0.08 (FC > 1.2 or FC < 0.83 [fold change, FC]) were defined as differentially expressed proteins (DEPs).

##### The Functional Analysis of Protein and DEP

Gene Ontology (GO) and InterPro (IPR) functional analysis were conducted using the interproscan program against the non-redundant protein database (including Pfam, PRINTS, ProDom, SMART, ProSite, and PANTHER), and the databases of COG (Clusters of Orthologous Groups) and KEGG (Kyoto Encyclopedia of Genes and Genomes) were used to analyze the protein family and pathway. DEPs were used for Volcanic map analysis, cluster heat map analysis, and enrichment analysis of GO and KEGG. The mass spectrometry proteomics data have been deposited to the ProteomeXchange Consortium (http://proteomecentral.proteomexchange.org (accessed on 14 October 2021)) via the iProX partner repository with the dataset identifier PXD043126.

### 4.4. Metabolomics

#### 4.4.1. Metabolites Extraction

Tissues (100 mg) were individually grounded with liquid nitrogen and the homogenate was resuspended with prechilled 80% methanol by well vortex. The samples were incubated on ice for 5 min and then were centrifuged at 15,000× *g*, 4 °C, for 20 min. Some of supernatant was diluted to final concentration containing 53% methanol by LC-MS-grade water. The samples were subsequently transferred to a fresh Eppendorf tube and then were centrifuged at 15,000× *g*, 4 °C, for 20 min. Finally, the supernatant was injected into the LC-MS/MS system analysis.

#### 4.4.2. UHPLC-MS/MS Analyses

UHPLC-MS/MS analyses were performed using a Vanquish UHPLC system (Thermo Fisher, Wilmington, DE, USA) coupled with an Orbitrap Q ExactiveTM HF mass spectrometer (Thermo Fisher, Wilmington, DE, USA) in Novogene Co., Ltd. (Beijing, China). Samples were injected onto a Hypesil Goldcolumn (100 × 2.1 mm, 1.9 μm) using a 17 min linear gradient at a flow rate of 0.2 mL/min. The eluents for the positive polarity mode were eluent A (0.1% FA in Water) and eluent B (Methanol). The eluents for the negative polarity mode were eluent A (5 mM ammonium acetate, pH 9.0) and eluent B (Methanol).The solvent gradient was set as follows: 2% B, 1.5 min; 2–85% B, 3 min; 100% B, 10 min; 100–2% B, 10.1 min; and 2% B, 12 min. Q ExactiveTM HF mass spectrometer was operated in positive/negative polarity mode with spray voltage of 3.5 kV, capillary temperature of 320 °C, sheath gas flow rate of 35 arb and aux gas flow rate of 10 arb, S-lens RF level of 60, and Aux gas heater temperature of 350 °C.

#### 4.4.3. Data Processing and Metabolite Identification

The raw data files generated by UHPLC-MS/MS were processed using the Compound Discoverer 3.1 (CD3.1, Thermo Fisher) to perform peak alignment, peak picking, and quantitation for each metabolite. After that, peak intensities were normalized to the total spectral intensity. The normalized data were used to predict the molecular formula based on additive ions, molecular ion peaks, and fragment ions. And then peaks were matched with the mzCloud (https://www.mzcloud.org/ (accessed on 12 October 2021)), mzVault, and MassList database to obtain the accurate qualitative and relative quantitative results. Statistical analyses were performed using the statistical software R (R version R-3.4.3), Python (Python 2.7.6 version), and CentOS (CentOS release 6.6).

#### 4.4.4. Data Analysis

These metabolites were annotated using the KEGG database (https://www.genome.jp/kegg/pathway.html (accessed on 14 October 2021)), HMDB database (https://hmdb.ca/metabolites (accessed on 14 October 2021)), and LIPIDMaps database (http://www.lipidmaps.org/ (accessed on 14 October 2021)). Principal components analysis (PCA) and Partial least squares discriminant analysis (PLS-DA) were performed at metaX [50] (a flexible and comprehensive software for processing metabolomics data).We applied univariate analysis (*t*-test) to calculate the statistical significance (*p*-value).The metabolites with VIP > 1, *p*-value < 0.05, and fold change ≥ 2 or FC ≤ 0.5 were considered to be differential metabolites. Volcano plots were used to filter metabolites of interest based on log_2_(FoldChange) and −log_10_(*p*-value) of metabolites by ggplot2 in R language.

For clustering heat maps, the data were normalized using z-scores of the intensity areas of differential metabolites and were plotted by Pheatmap package in R language. The correlations between differential metabolites were analyzed by cor () in R language (method = Pearson). Statistical significance of correlations between differential metabolites was calculated by cor.mtest() in R language. *p*-value < 0.05 was considered as statistically significant and correlation plots were plotted by corrplot package in R language. The functions of these metabolites and metabolic pathways were studied using the KEGG database. The metabolic pathways enrichment of differential metabolites was performed. When ratios were satisfied by x/n > y/N, metabolic pathways were considered as having enrichment; when *p*-value of metabolic pathway <0.05, metabolic pathways were considered as having statistically significant enrichment.

### 4.5. Hematoxylin and Eosin (H&E) Staining

Liver and brain specimens from the different treatment groups were fixed in 4% paraformaldehyde for 24 h, embedded in paraffin after being washed in PBS, dehydrated in gradient alcohol, dealcoholized in xylene, and then sliced into 5 µm sections. Sections were stained using standard H&E staining methods and examined by two blinded pathologists. Finally, the sections were mounted with neutral gum and captured by an Olympus microscope (Olympus Corp., Tokyo, Japan).

### 4.6. Determination of ALT, AST, MDA, and GSH

Levels of ALT, AST, MDA, and GSH in serum samples were evaluated using detection kits, according to the manufacturer’s instructions.

### 4.7. RNA Extraction and Quantitative Real-Time PCR (qPCR)

RNAex Pro Reagent (AG; 21102) was used to extract total RNA, as indicated by the supplier, and the Reverse Transcription Premix kit (AG; 11728) was used to produce cDNA following the manufacturer’s recommendations. Quantitative real-time PCR was performed using SYBR Green Pro Taq HS qPCR kit (AG; 11701) and LightCycler480 (Roche, Mannheim, Germany). Actin was used as the internal standard control to normalize gene expression using the 2^−ΔΔCt^ method. The sequences of the qPCR primers are listed in Supplementary Table S2.

### 4.8. Statistical Analysis

ALT, AST, MDA, and GSH levels were presented as means ± standard deviation (SD) and analyzed using IBM SPSS Statistics for Macintosh, Version 26.0 (SPSS Inc., Chicago, IL, USA). Student’s test was used if a significant difference was determined. *p* < 0.05 was considered statistically significant.

## 5. Conclusions

In conclusion, the mechanism of hepatic encephalopathy may be correlated to the energy metabolism, amino acid metabolism, complement and coagulation cascades, vitamin digestion and absorption, and neurotransmitter imbalance in this experiment, based on the metabolomics and proteomics. More importantly, we found that the mechanism of hepatic encephalopathy may be related to the ferroptosis pathway. However, the specific pathway and mechanism of differential expression proteins still need to be further studied. Meanwhile, the sample size of this study need to be increased.

## Figures and Tables

**Table 1:** Significant differential metabolites between the HE group and the control group.

No.	Name	log_2_FC	*p* Value	ROC	VIP	Up/Down
1	L-Glutamic acid	−0.7148	0.0000	1.0000	1.7550	down
2	Adenine	−1.8026	0.0003	1.0000	1.4061	down
3	Adenosine	−4.2551	0.0011	1.0000	1.3706	down
4	Cytidine	−1.3588	0.0256	0.8611	1.0604	down
5	L-Glutathione (reduced)	−2.0501	0.0288	0.9167	1.0914	down
6	UDP-N-acetylglucosamine	−1.5685	0.0290	0.8611	1.1164	down
7	S-Adenosylmethionine	−1.8971	0.0177	1.0000	1.1697	down
8	N-Acetyl-DL-glutamic acid	−0.9836	0.0323	0.7778	1.1143	down
9	5′-S-Methyl-5′-thioadenosine	−1.2561	0.0350	0.7222	1.1118	down
10	Υ-Glutamylcysteine	−1.1471	0.0426	0.7222	1.0854	down
11	5-Hydroxyindole-3-acetic acid	−0.8199	0.0470	0.7778	1.0177	down
12	Cytidine 5′-monophosphate (hydrate)	−0.8442	0.0475	0.8056	1.0850	down
13	Inosine	−0.7601	0.0481	0.7778	1.0709	down
14	α-Aspartylphenylalanine	−2.4091	0.0482	0.8056	1.1948	down
15	L-Glutathione oxidized	−1.1454	0.0490	0.8333	1.0628	down
16	α-D-Glucose-1,6-bisphosphate	−1.6690	0.0388	0.8333	1.0271	down
17	N-Acetylneuraminic acid	−0.5952	0.0001	1.0000	1.4531	down
18	5-Aminolevulinic acid	−0.9738	0.0017	1.0000	1.2759	down
19	Adenosine 5′-monophosphate	−3.0832	0.0023	0.9444	1.2062	down
20	Ascorbic acid	−0.9980	0.0129	0.8889	1.1806	down
21	O-Phosphorylethanolamine	−1.0382	0.0368	0.9444	1.0457	down
22	Uridine monophosphate (UMP)	−1.6295	0.0232	0.8611	1.1090	down
23	Guanosine monophosphate (GMP)	−1.6259	0.0234	0.9167	1.0563	down
24	N6-Acetyl-L-lysine	0.5880	0.0499	0.8333	1.0435	up
25	N-Acetyl-L-tyrosine	2.1337	0.0158	0.9167	1.1105	up
26	L-Histidine	2.2341	0.0065	1.0000	1.2401	up
27	Hippuric acid	2.6338	0.0273	0.8611	1.0633	up
28	N2-Methylguanosine	2.7800	0.0330	0.7222	1.0859	up
29	Xanthosine	4.1746	0.0179	0.8611	1.1467	up
30	N-Acetyl-L-phenylalanine	2.1029	0.0189	0.9167	1.1265	up
31	L-Tyrosine	1.2616	0.0443	0.7222	1.0675	up
32	N-Acetyl-DL-tryptophan	1.7116	0.0223	0.8333	1.0564	up
33	Argininosuccinic acid	1.0689	0.0018	0.9444	1.2466	up
34	N3,N4-Dimethyl-L-arginine	2.6446	0.0020	0.9722	1.3649	up
35	L-Phenylalanine	1.4515	0.0031	1.0000	1.3137	up
36	Uracil	1.8273	0.0047	1.0000	1.2165	up
37	DL-Tryptophan	1.4018	0.0062	1.0000	1.2632	up
38	L(+)-Ornithine	3.8581	0.0063	0.8611	1.2678	up
39	Proline	3.7575	0.0063	0.8611	1.2684	up
40	γ-aminobutyric acid	2.3444	0.0067	1.0000	1.2419	up
41	Thiamine	0.9335	0.0081	0.9444	1.1754	up
42	Indole-3-acetic acid	1.0212	0.0089	0.9167	1.1264	up
43	Nicotinamide	3.0732	0.0388	0.7222	1.0653	up
44	DL-Lysine	2.0674	0.0141	0.9167	1.1979	up
45	L-Isoleucine	1.7751	0.0158	0.9444	1.1804	up
46	L-(+)-Citrulline	3.7701	0.0245	0.8611	1.1390	up
47	Threonine	0.8236	0.0246	0.8333	1.0965	up
48	N-Acetyl-L-leucine	2.3822	0.0442	0.7222	1.0795	up
49	Riboflavin	1.9154	0.0260	0.8611	1.0731	up
50	Leukotriene B4	1.0947	0.0405	0.8333	1.0335	up

**Table 2:** Peptide fraction separation liquid chromatography elution gradient table.

Time (min)	Flow Rate (mL/min)	Mobile Phase A (%)	Mobile Phase B (%)
0	1	97	3
10	1	95	5
30	1	80	20
48	1	60	40
50	1	50	50
53	1	30	70
54	1	0	100

**Table 3:** Liquid chromatography elution gradient table.

Time (min)	Flow Rate (mL/min)	Mobile Phase A (%)	Mobile Phase B (%)
0	0.6	94	6
2	0.6	85	15
48	0.6	60	40
50	0.6	50	50
51	0.6	45	55
60	0.6	0	100

## Data Availability

All study data are included in the article and Supplementary Materials.

## Supplementary Materials

The following supporting information can be downloaded at: https://www.mdpi.com/article/10.3390/ijms25010284/s1.

## Abbreviations

ACN	acetonitrile
AGC	automatic gain control
ALT	alanine transaminase
AST	aspartate aminotransferase
BCAAs	branched chain amion acids
BP	biological Process
CC	Cellular Component
COG	clusters of orthologous groups
CYP450	cytochrome P450
DEPs	differentially expressed proteins
ELISA	enzyme-linked immunosorbent assay
FA	formic acid
FC	fold change
FMO	flavin-containing monooxygenase
FMT	fecal microbiota transplantation
FTH1	ferritin heavy chain 1
GABA	γ-aminobutyric acid
Glu	glutamate
GMP	guanosine monophosphate
GO	gene ontology
GPX4	glutathione peroxidase 4
GSH	glutathione
HCD	higher-energy collisional dissociation
HE	hepatic encephalopathy
HRMS	high-resolution mass spectrometry
KEGG	Kyoto Encyclopedia of Genes and Genomes
LC-MS	Liquid chromatography–Mass spectrometry
LOLA	L-ornithine-L-aspartate
MDA	malondialdehyde
MeOH	Methanol
MF	Molecular Function
NAFLD	nonalcoholic fatty liver disease
NCOA4	nuclear receptor coactivator 4
PBS	phosphate-buffered saline
PCA	principal component analysis
PLS-DA	partial least squares discrimination analysis
PSMs	peptide spectrum matches
PUFA	polyunsaturated fatty acid
QC	quality control
ROS	reactive oxygen species
SEM	standard error of mean
SPF	specific pathogen-free
STEAP3	six-transmembrane epithelial antigen of prostate 3
TAA	thioacetamide
TMT	tandem mass tags
UMP	uridine monophosphate
VIP	variable importance in projection
