# Supplementary material for: The Mechanism of Hepatic Encephalopathy Induced by Thioacetamide Based on Metabolomics and Proteomics: A Preliminary Study
Source: Int J Mol Sci. 2023 Dec 24;25(1):284. doi: 10.3390/ijms25010284 (PMC10779174; doi:10.3390/ijms25010284)
Supplement: Supplementary file 1 [file ijms-25-00284-s001.zip › ijms-2749916-supplementary.pdf]

International Journal of *Molecular Sciences*

MDPI

Communication

The Mechanism of Hepatic Encephalopathy Induced by Thioacetamide Based on Metabolomics and Proteomics: A Preliminary Study

Honghui Guo [1,2,3,†], Guang Wang [4,†], Wei Huang [1,2,3], Lingrui Li [1,2,3], Yang Bai [1,2,3], Haifeng Wang [1,2,3] and Lina Gao [1,2,3,*]

[1] Liaoning Province Key Laboratory of Forensic Bio-Evidence Sciences, Shenyang 110122, China; 18856834146@163.com (H.G.); vivihuang98@163.com (W.H.); lilingrui1999@163.com (L.L.); 13840852382@163.com (Y.B.); anaroberts195@gmail.com (H.W.)
[2] China Medical University Center of Forensic Investigation, Shenyang 110122, China
[3] Department of Forensic Analytical Toxicology, China Medical University, Shenyang 110122, China
[4] Department of Laboratory Animal Science, China Medical University, Shenyang 110122, China; gwang@cmu.edu.cn
* Correspondence: lngao@cmu.edu.cn; Tel.: +86-189-0091-0140
[†] These authors contributed equally to this work.

Supplementary Materials

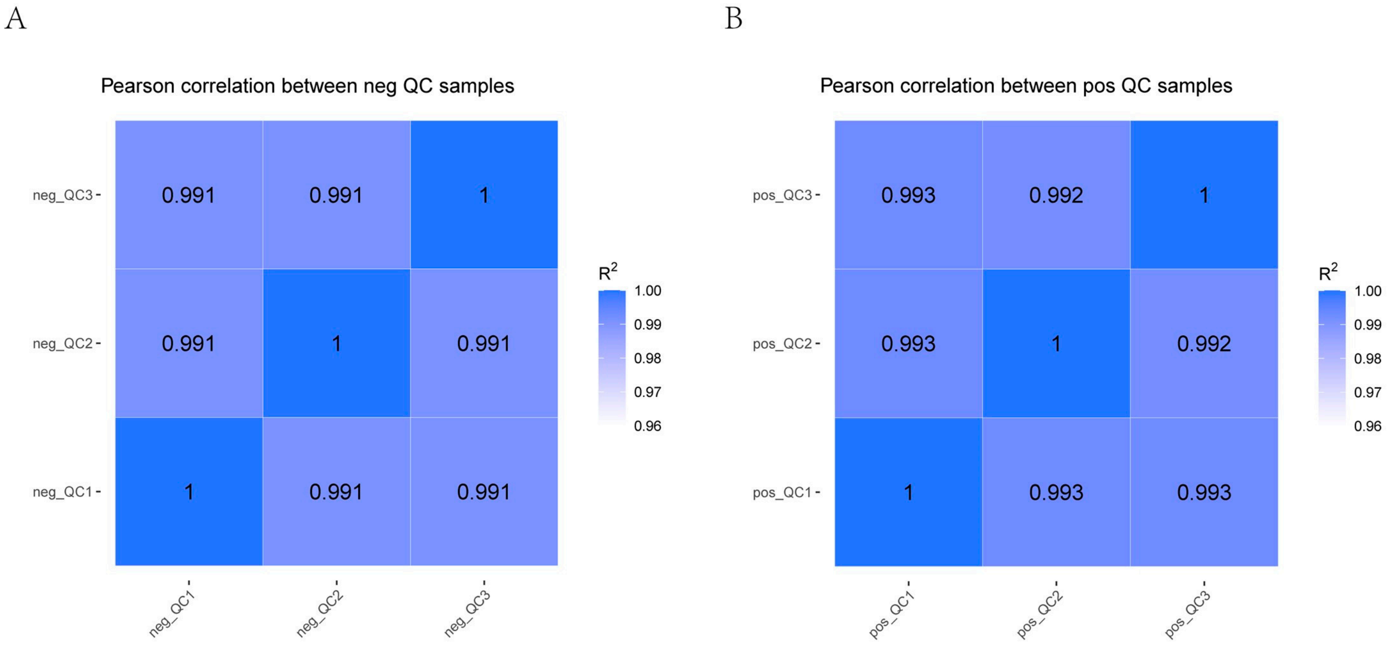

Supplementary Figure S1. Pearson correlation between QC samples (the left one was obtained in the negative polarity mode, and the right one was obtained in the positive polarity mode**).**

Supplementary Table S1. The differential expressed proteins between the HE group and the control group.

No.	Protein	Description	S.vs.C FC	S.vs.C Pvalue	S.vs.C log2FC	S.vs.C UP.DOWN
1	B2RPW2	Smith-Magenis syndrome chromosome region, candidate 7-like (Human)	1.2482	0.0006	0.3199	up
2	Q3UXG7	GDP-fucose protein O-fucosyltransferase 1	1.3222	0.0009	0.4030	up
3	P32233	Developmentally-regulated GTP-binding protein 1	1.2441	0.0010	0.3151	up
4	Q2M4H7	E3 ubiquitin-protein ligase CBL	0.5743	0.0014	-0.800	down

5	Q3TDV5	Uncharacterized protein	1.2106	0.0014	0.2757	up
6	Q8K0S5	Reticulon-4 receptor-like 1	1.2070	0.0016	0.2715	up
7	O08677	Kininogen-1	1.2962	0.0016	0.3743	up
8	Q99P72	Reticulon-4	0.7849	0.0019	-0.349	down
9	A0A286 YDH6	Calcium-dependent secretion activator 1	0.8073	0.0022	-0.309	down
10	P21614	Vitamin D-binding protein	1.2823	0.0022	0.3587	up
11	E0CXQ1	High affinity cAMP-specific and IBMX-insensitive 3,5-cyclic phosphodiesterase 8B (Fragment)	0.7529	0.0024	-0.409	down
12	Q8C4R2	cGMP-dependent protein kinase	0.7072	0.0030	-0.500	down
13	B0FTY3	NudC-like protein	0.7524	0.0032	-0.410	down
14	A0A6M8 TZJ8	ATP synthase subunit a	1.4468	0.0033	0.5329	up
15	Q8C561	G-protein coupled receptor-associated protein LMBRD2	0.8298	0.0035	-0.269	down
16	D3Z3F8	Spastic paraplegia 20, spartin (Troyer syndrome) homolog (human)	0.8084	0.0036	-0.307	down
17	E9PUA3	IQ motif and SEC7 domain-containing protein 1	0.7615	0.0037	-0.393	down
18	Q5U430	E3 ubiquitin-protein ligase UBR3	0.8292	0.0037	-0.270	down
19	Q8VDZ4	Palmitoyltransferase ZDHHC5	0.5818	0.0039	-0.781	down
20	O35945	Aldehyde dehydrogenase, cytosolic 1	1.3535	0.0041	0.4368	up
21	E9PV26	Proline-rich 36	0.8159	0.0042	-0.293	down
22	Q7M750	Opalin	0.5527	0.0043	-0.855	down
23	Q3V2C6	IF rod domain-containing protein	1.2587	0.0044	0.3320	up
24	O88704	Potassium/sodium hyperpolarization-activated cyclic nucleo-tide-gated channel 1	0.8164	0.0045	-0.293	down
25	Q9JIG4	Protein phosphatase 1 regulatory subunit 3F	0.6713	0.0045	-0.575	down
26	F8WGR0	Alpha-adducin	0.6776	0.0048	-0.561	down
27	P06728	Apolipoprotein A-IV	1.3567	0.0049	0.4402	up
28	Q4VAA2	Protein CDV3	0.7621	0.0054	-0.392	down
29	Q330P7	SCF apoptosis response protein 1	0.7234	0.0057	-0.467	down
30	Q3TVY3	Uncharacterized protein	1.2782	0.0059	0.3542	up
31	Q3TIB7	Gamma-tubulin complex component	1.4380	0.0060	0.5241	up
32	Q7TSJ2	Microtubule-associated protein 6	0.7710	0.0060	-0.375	down
33	Q3TR92	Ion_trans domain-containing protein	0.5795	0.0063	-0.7870	down
34	P63054	Calmodulin regulator protein PCP4	0.6648	0.0063	-0.589	down
35	Q2UZW7	Microtubule-associated protein	0.7623	0.0063	-0.392	down
36	Q3UBT6	Protein kinase domain-containing protein	0.7111	0.0064	-0.492	down
37	Q9ET77	Junctophilin-3	0.7357	0.0066	-0.443	down
38	Q571F3	MFLJ00061 protein (Fragment)	0.8269	0.0069	-0.274	down
39	Q3UZP4	Small VCP/p97-interacting protein	0.7803	0.0071	-0.358	down
40	Q8K2F8	Protein LSM14 homolog A	0.8022	0.0071	-0.318	down
41	Q8R1T1	Charged multivesicular body protein 7	0.8081	0.0072	-0.307	down
42	A2AJI1	MAP7 domain-containing protein 1	0.6431	0.0076	-0.637	down
43	P22599	Alpha-1-antitrypsin 1-2	1.6445	0.0076	0.7177	up
44	Q921I1	Serotransferrin	1.4633	0.0076	0.5493	up
45	Q9JM96	Cdc42 effector protein 4	0.7358	0.0077	-0.442	down
46	Q6NS82	Reticulophagy regulator 2	0.7818	0.0077	-0.355	down
47	Q9CWE0	Mitochondrial fission regulator 1-like	0.7781	0.0078	-0.362	down
48	Q8BNA5	SH3 domain-containing protein	0.6565	0.0078	-0.607	down
49	Q9QYX7	Protein piccolo	0.8049	0.0080	-0.313	down
50	A0A087 WQ92	Histone deacetylase 4 (Fragment)	0.7136	0.0081	-0.487	down
51	V9GXD9	Synergin gamma	0.7784	0.0081	-0.361	down

52	Q9ERG2	Striatin-3	0.8205	0.0082	-0.285	down
53	Q91ZZ3	Beta-synuclein	0.6520	0.0084	-0.617	down
54	Q5U3K5	Rab-like protein 6	0.8315	0.0086	-0.266	down
55	O08539	Myc box-dependent-interacting protein 1	0.8090	0.0087	-0.306	down
56	P49070	Guided entry of tail-anchored proteins factor CAMLG	0.6920	0.0087	-0.531	down
57	Q546G4	Serum albumin	1.7843	0.0090	0.8354	up
58	Q9CZN4	Protein shisa-9	0.7008	0.0091	-0.513	down
59	Q8C181	Muscleblind-like protein 2	0.8274	0.0092	-0.273	down
60	Q7TQG1	Pleckstrin homology domain-containing family A member 6	0.8221	0.0093	-0.282	down
61	Q00623	Apolipoprotein A-I	1.4901	0.0095	0.5754	up
62	G3UZ30	E3 ubiquitin-protein ligase PPP1R11 (Fragment)	0.4652	0.0095	-1.104	down
63	O55047	Serine/threonine-protein kinase tousled-like 2	0.7889	0.0095	-0.342	down
64	A0A668 KLC6	Microtubule-associated protein	0.6582	0.0097	-0.603	down
65	A0A2R8 W6S4	Protein kinase C and casein kinase substrate in neurons protein 2 (Fragment)	0.5365	0.0097	-0.898	down
66	Q99JX6	Annexin	1.4250	0.0098	0.5110	up
67	E9PUC5	PH and SEC7 domain-containing protein 3	0.6608	0.0098	-0.598	down
68	Q4FJL2	Reticulon	0.5887	0.0099	-0.764	down
69	G3X8Q5	Ceruloplasmin	1.4267	0.0101	0.5128	up
70	A2ARP8	Microtubule-associated protein 1A	0.7194	0.0101	-0.475	down
71	A0A668 KLV9	A-kinase anchor protein 12	0.7223	0.0103	-0.469	down
72	P01027	Complement C3	1.2231	0.0103	0.2906	up
73	Q6PEV3	WAS/WASL-interacting protein family member 2	0.6808	0.0104	-0.555	down
74	Q8VD12	Zinc finger protein 385A	0.7434	0.0106	-0.428	down
75	Q3UNH4	G protein-regulated inducer of neurite outgrowth 1	0.7512	0.0106	-0.413	down
76	D0VYV6	Band 4.1-like protein 3	0.8311	0.0106	-0.267	down
77	H3BLG8	Protein CUSTOS (Fragment)	0.5062	0.0108	-0.982	down
78	Q9ES97	Reticulon-3	0.7044	0.0110	-0.505	down
79	E9Q1K3	Alpha-adducin	0.7134	0.0111	-0.487	down
80	Q8R318	2310005E10Rik protein	1.2351	0.0112	0.3047	up
81	Q3TDD8	RRM domain-containing protein	0.8221	0.0113	-0.283	down
82	E9Q7M2	TSC22 domain family, member 2	0.7663	0.0114	-0.384	down
83	Q8VD37	SH3-containing GRB2-like protein 3-interacting protein 1	0.8256	0.0117	-0.276	down
84	O88271	Craniofacial development protein 1	0.7919	0.0120	-0.337	down
85	A0A1B0 GT88	Brain-enriched guanylate kinase-associated protein	0.7198	0.0120	-0.474	down
86	Q71M36	Chondroitin sulfate proteoglycan 5	0.7995	0.0124	-0.323	down
87	A0A0R4J 2A2	Ankyrin repeat and sterile alpha motif domain-containing protein 1B	0.3357	0.0125	-1.575	down
88	S4R1C4	Calcium-transporting ATPase	0.7818	0.0129	-0.355	down
89	Q78YZ6	Short coiled-coil protein	0.6978	0.0131	-0.519	down
90	P14873	Microtubule-associated protein 1B	0.8208	0.0132	-0.285	down
91	Q9QXF8	Glycine N-methyltransferase	1.3566	0.0133	0.4400	up
92	Q9Z0P4	Paralemmin-1	0.8076	0.0135	-0.308	down
93	Q6A065	Centrosomal protein of 170 kDa	0.7881	0.0135	-0.344	down
94	Q6ZQ68	MKIAA0687 protein (Fragment)	0.7257	0.0136	-0.462	down
95	A0A0N4 SV74	WASH complex subunit 2 (Fragment)	0.8180	0.0136	-0.290	down
96	P04247	Myoglobin	2.0111	0.0139	1.0080	up
97	A0A7N9 VR94	AHNAK nucleoprotein 2	0.6404	0.0139	-0.643	down

98	Q80W47	WD repeat domain phosphoinositide-interacting protein 2	0.8236	0.0140	-0.280	down
99	Q9DC07	LIM zinc-binding domain-containing Nebulette	0.7755	0.0141	-0.367	down
100	F6RRH9	Dynamin-1 (Fragment)	0.5399	0.0142	-0.889	down
101	O08919	Numb-like protein	0.8297	0.0142	-0.269	down
102	Q8CCJ4	APC membrane recruitment protein 2	0.7644	0.0143	-0.387	down
103	Q8CH25	SAFB-like transcription modulator	0.7869	0.0144	-0.346	down
104	Q76N33	AMSH-like protease	0.7805	0.0144	-0.357	down
105	Q80U23	Syntaphilin	0.7292	0.0147	-0.455	down
106	Q8K1M6	Dynamin-1-like protein	0.8126	0.0148	-0.299	down
107	A0A0A0 MQC7	Microtubule-associated protein	0.7644	0.0148	-0.387	down
108	Q8CBM0	Uncharacterized protein	0.7518	0.0151	-0.412	down
109	Q5DTJ4	MKIAA4178 protein (Fragment)	0.7907	0.0151	-0.339	down
110	Q8C3W1	Uncharacterized protein C1orf198 homolog	0.7693	0.0152	-0.378	down
111	Q3UHE3	Ig-like domain-containing protein	1.2827	0.0157	0.3592	up
112	Q9JMK2	Casein kinase I isoform epsilon	0.7714	0.0157	-0.374	down
113	B1AXZ5	ELAV-like protein	0.7248	0.0157	-0.464	down
114	E9Q3E3	Glycine receptor subunit beta	0.8121	0.0158	-0.300	down
115	P23953	Carboxylesterase 1C	1.2878	0.0158	0.3649	up
116	I7HJR3	Beta-2-glycoprotein 1 (Fragment)	1.2463	0.0159	0.3177	up
117	A0A1D5 RL96	BTB/POZ domain-containing protein 8	0.6560	0.0162	-0.608	down
118	Q91X72	Hemopexin	1.4401	0.0165	0.5262	up
119	O88737	Protein bassoon	0.7582	0.0166	-0.399	down
120	Q9R0L7	A-kinase anchor protein 8-like	1.3166	0.0167	0.3969	up
121	O55042	Alpha-synuclein	0.7535	0.0168	-0.408	down
122	Q3UWT7	Phospholipase D (Fragment)	1.2321	0.0169	0.3012	up
123	A8DUP7	Beta-globin	1.5921	0.0170	0.6710	up
124	Q61838	Pregnancy zone protein	1.3931	0.0171	0.4784	up
125	A2RRY2	2310046A06Rik protein	0.7133	0.0172	-0.487	down
126	P07758	Alpha-1-antitrypsin 1-1	2.2548	0.0173	1.1730	up
127	Q9D1J3	SAP domain-containing ribonucleoprotein	0.6887	0.0179	-0.538	down
128	Q9ERU3	Zinc finger protein 22	0.7949	0.0180	-0.331	down
129	Q5EBJ4	Ermin	0.7331	0.0188	-0.448	down
130	Q543N3	LIM and SH3 domain protein 1	0.7048	0.0189	-0.505	down
131	Q9QYK7	RING finger protein 11	0.8064	0.0193	-0.310	down
132	E9Q8N8	Anion exchange protein	0.7061	0.0195	-0.502	down
133	P06909	Complement factor H	1.2473	0.0195	0.3189	up
134	Q3U007	Uncharacterized protein	0.7488	0.0199	-0.417	down
135	A0A0B4J 1E2	SNW domain-containing protein 1	0.7987	0.0201	-0.324	down
136	D3YXK2	Scaffold attachment factor B1	0.8226	0.0203	-0.282	down
137	D3YVR9	RIKEN cDNA 1110004F10 gene	0.7873	0.0205	-0.345	down
138	A0A2I3B RM6	Heterogeneous nuclear ribonucleoproteins C1/C2 (Fragment)	0.7065	0.0206	-0.501	down
139	A2ANL1	Solute carrier family 23 member 2 (Fragment)	0.6268	0.0207	-0.674	down
140	F7AA26	Paralemmin A kinase anchor protein (Fragment)	0.7950	0.0209	-0.331	down
141	Q8K232	Adducin 1 (Alpha)	0.8119	0.0210	-0.301	down
142	B5TVM2	Immunoglobulin-like domain-containing receptor 2	0.8302	0.0210	-0.268	down
143	Q8BR92	Paralemmin-2	0.7539	0.0212	-0.407	down
144	Q9JKS5	Intracellular hyaluronan-binding protein 4	0.8284	0.0215	-0.272	down
145	P27546	Microtubule-associated protein 4	0.8073	0.0216	-0.309	down
146	G3X9G2	Misshapen-like kinase 1	0.7219	0.0217	-0.470	down

147	Q8CGC4	Protein LSM14 homolog B	0.6682	0.0221	-0.581	down
148	P13634	Carbonic anhydrase 1	1.2815	0.0226	0.3579	up
149	P97825	Jupiter microtubule associated homolog 1	0.7545	0.0226	-0.406	down
150	A0A411P8U6	Myelin basic protein (Fragment)	0.7495	0.0228	-0.416	down
151	Q8K327	Chromosome alignment-maintaining phosphoprotein 1	0.6621	0.0229	-0.595	down
152	Q542T4	Myelin basic protein	0.6550	0.0231	-0.610	down
153	B2RWU4	Cordon-bleu	0.7628	0.0234	-0.391	down
154	Q4FJM2	Cyclin-dependent kinase inhibitor 1B	0.8173	0.0244	-0.291	down
155	B7ZNJ3	Caskin1 protein	0.8085	0.0247	-0.307	down
156	A0A0R4J1G9	Metalloreductase STEAP3	1.2113	0.0248	0.2766	up
157	Q9CU36	Protein kinase domain-containing protein (Fragment)	0.7963	0.0249	-0.329	down
158	Q80UP8	Sodium-dependent phosphate transporter 2	0.7303	0.0249	-0.453	down
159	E9Q1G8	Septin	0.7974	0.0251	-0.327	down
160	A0A654ICL5	Connexin p1	0.8195	0.0252	-0.287	down
161	Q3UHN4	Voltage-dependent P/Q-type calcium channel subunit alpha	0.8188	0.0252	-0.288	down
162	Q9WV69	Dematin	0.7942	0.0253	-0.332	down
163	A0A3B2WCD2	Serine/threonine-protein kinase LMTK3	0.7762	0.0253	-0.365	down
164	Q3ULL6	UPF3 regulator of nonsense transcripts homolog B (yeast)	0.6153	0.0255	-0.701	down
165	P01872	Immunoglobulin heavy constant mu	1.2060	0.0255	0.2703	up
166	Q8BG13	RNA-binding protein 3	0.7972	0.0265	-0.327	down
167	S4R1Y1	Cullin-9	0.6676	0.0279	-0.583	down
168	Q8BNE1	TRPM8 channel-associated factor 1	1.5458	0.0282	0.6284	up
169	C0HKD9	Microfibrillar-associated protein 1B	0.8283	0.0287	-0.272	down
170	D3Z5K8	SH3 and multiple ankyrin repeat domains protein 2	0.7727	0.0289	-0.372	down
171	D3YX76	Glutathione S-transferase	1.4115	0.0291	0.4972	up
172	Q99P69	Kinetochore protein Nuf2	0.7121	0.0306	-0.490	down
173	Q5M9P7	Calcium regulated heat stable protein 1	0.8298	0.0307	-0.269	down
174	Q3TDE4	Nicastrin (Fragment)	1.2021	0.0307	0.2656	up
175	Q3TNL6	Anion exchange protein	1.3543	0.0309	0.4376	up
176	P07759	Serine protease inhibitor A3K	1.6049	0.0309	0.6825	up
177	A0A0J9YTU3	Cytospin-B	0.8295	0.0317	-0.270	down
178	Q3T1F6	Myelin-associated glycoprotein	0.5812	0.0317	-0.783	down
179	Q3UI81	Uncharacterized protein	0.8053	0.0320	-0.312	down
180	Q14BB9	MAP6 domain-containing protein 1	0.7932	0.0321	-0.334	down
181	A0A087WPF5	AN1-type zinc finger protein 2B (Fragment)	0.7558	0.0322	-0.404	down
182	F7B1A4	Mitochondrial fission factor (Fragment)	0.6561	0.0325	-0.608	down
183	Q69ZQ5	MKIAA1150 protein (Fragment)	0.7718	0.0327	-0.374	down
184	E9PYD9	Folate receptor alpha (Fragment)	1.4730	0.0327	0.5588	up
185	Q3TM89	PEST proteolytic signal-containing nuclear protein	0.8264	0.0332	-0.275	down
186	Q68ED7	CREB-regulated transcription coactivator 1	0.8171	0.0341	-0.291	down
187	Q810M8	Ubiquitinyl hydrolase 1 (Fragment)	0.8082	0.0342	-0.307	down
188	Q05D14	Clmn protein (Fragment)	0.8045	0.0343	-0.314	down
189	Q80YN3	Breast carcinoma-amplified sequence 1 homolog	0.8088	0.0344	-0.306	down
190	E9PZ43	Microtubule-associated protein	0.7043	0.0344	-0.506	down
191	Q8BHL7	CDC42 small effector protein 1	0.7557	0.0344	-0.404	down
192	Q9QUN9	Dickkopf-related protein 3	1.2011	0.0353	0.2645	up
193	Q3TSA8	Secretory carrier-associated membrane protein	0.7858	0.0354	-0.348	down

194	Q9DB90	Protein SMG9	0.7969	0.0357	-0.327	down
195	Q3TIU3	Alpha-2-HS-glycoprotein	1.3145	0.0357	0.3946	up
196	Q6QWF9	Calcium/calmodulin-dependent protein kinase II inhibitor 1	0.7942	0.0361	-0.332	down
197	Q9CY06	GLOBIN domain-containing protein	1.5553	0.0364	0.6372	up
198	Q2VPR3	2500003M10Rik protein (Fragment)	0.7723	0.0366	-0.373	down
199	A0A0N4 SUV3	Vesicle-associated membrane protein 1 (Fragment)	0.8090	0.0369	-0.306	down
200	Q54AC6	Cox17p	0.8325	0.0371	-0.264	down
201	P60761	Neurogranin	0.4650	0.0372	-1.104	down
202	Q91VH6	Protein MEMO1	1.2144	0.0376	0.2803	up
203	Q08EE0	2310022M17Rik protein	0.7489	0.0377	-0.417	down
204	A0A0F7 QZE4	MAb 110 heavy chain	1.3882	0.0382	0.4732	up
205	A0A0A6 YW53	Mitogen-activated protein kinase kinase kinase kinase 4	0.6543	0.0393	-0.612	down
206	A2A841	Band 4.1	0.7993	0.0394	-0.323	down
207	A2AG50	MAP7 domain-containing protein 2	0.7927	0.0401	-0.335	down
208	A0A087 WQF0	Phospholipid phosphatase-related protein type 2	0.8155	0.0404	-0.295	down
209	Q3UFS0	Protein zyg-11 homolog B	1.2046	0.0417	0.2686	up
210	D3Y273	Anion exchange protein	0.8163	0.0418	-0.293	down
211	P08551	Neurofilament light polypeptide	0.8303	0.0421	-0.268	down
212	Q9JIX8	Apoptotic chromatin condensation inducer in the nucleus	0.8301	0.0422	-0.269	down
213	P28665	Murinoglobulin-1	1.5118	0.0423	0.5963	up
214	E9Q3E2	Synaptopodin	0.6568	0.0423	-0.606	down
215	Q9EQQ2	Protein YIPF5	0.7545	0.0425	-0.406	down
216	P60898	DNA-directed RNA polymerase II subunit RPB9	0.8186	0.0430	-0.289	down
217	B2RQS1	Striatin-3	0.8247	0.0431	-0.278	down
218	O55131	Septin-7	0.7237	0.0433	-0.466	down
219	A8DUK4	Beta-globin	1.5222	0.0460	0.6062	up
220	Q66JR8	Ptms protein	0.7999	0.0462	-0.322	down
221	P63040	Complexin-1	0.8288	0.0472	-0.271	down
222	P40936	Indolethylamine N-methyltransferase	1.6576	0.0476	0.7291	up
223	P20917	Myelin-associated glycoprotein	0.7251	0.0479	-0.464	down
224	Q8BWS5	G protein-regulated inducer of neurite outgrowth 3	0.7103	0.0492	-0.493	down
225	A3KMP2	Tetratricopeptide repeat protein 38	1.2536	0.0496	0.3261	up
226	Q6P1H9	Syt6 protein	0.7067	0.0496	-0.501	down

Supplementary Table S2. Quantitative reverse transcription PCR primer sequences.

Gene	Primer sequence	Species
SLC7A11-F	TCCTGCTTTGGCTCCATGAACG	Mouse
SLC7A11-R	AGAGGAGTGTGCTTGCGGACAT	Mouse
GPX4-F	ACAAGAACGGCTGCGTGGTGAA	Mouse
GPX4-R	GCCACACACTTGTGGAGCTAGA	Mouse
FTH-F	CCATCAACCGCCAGATCAAC	Mouse
FTH-R	GAAACATCATCTCGGTCAAA	Mouse
ACTB-F	GGCACCACACCTTCTACAACGAG	Mouse
ACTB-R	TCATCTTCTCACGGTTGGCTTTGG	Mouse
